# Supplementary figures and images for: eQTL mapping in transgenic alpha-synuclein carrying Caenorhabditis elegans recombinant inbred lines
Source: Hum Mol Genet. 2024 Oct 23;33(24):2123–32. doi: 10.1093/hmg/ddae148 (PMC11630767; doi:10.1093/hmg/ddae148)

**A**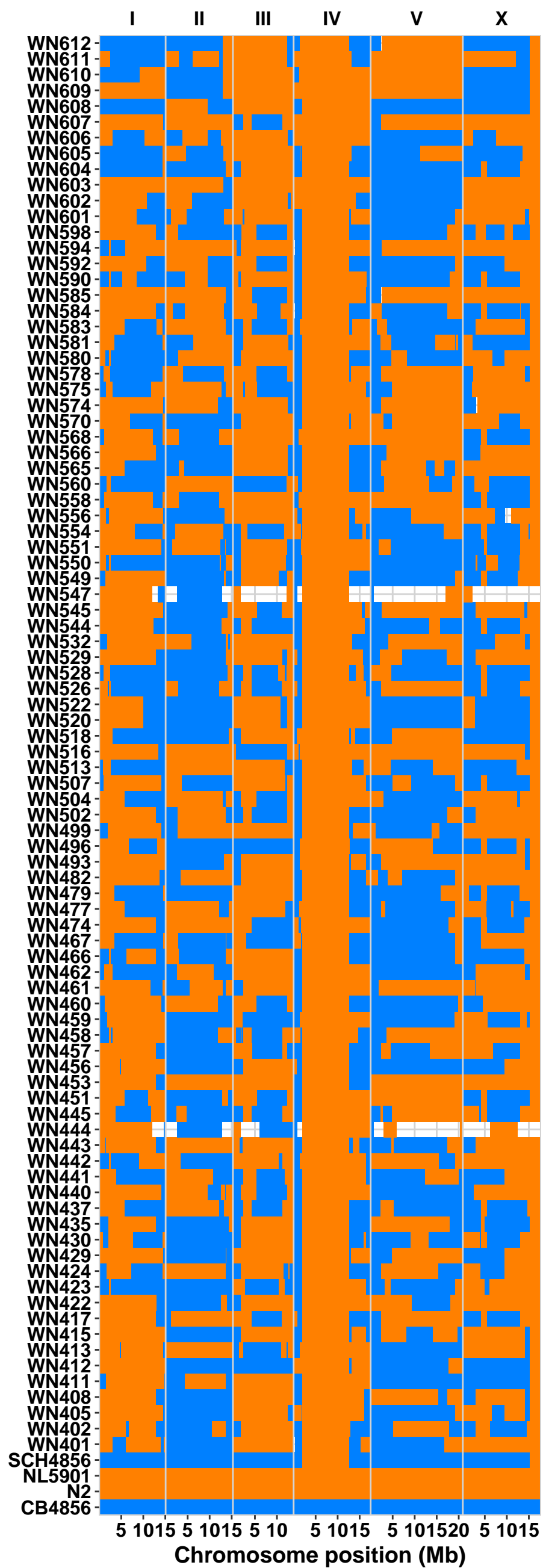**B**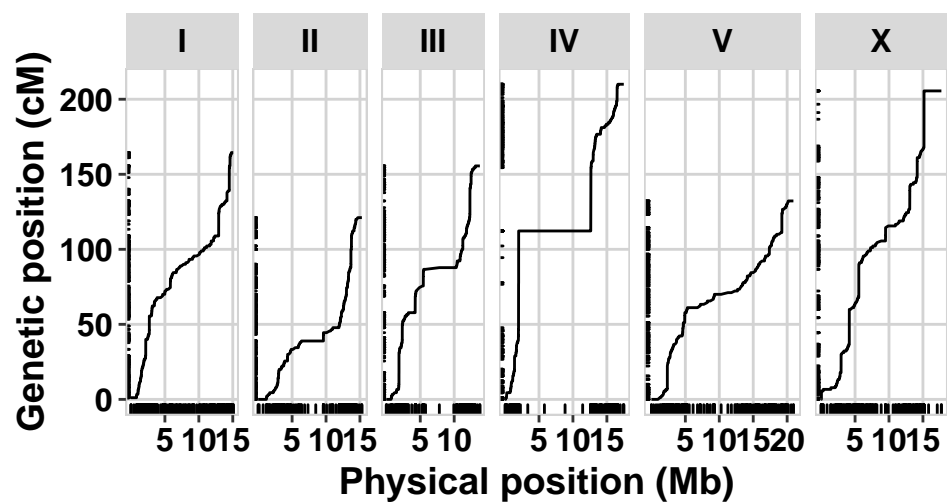**C**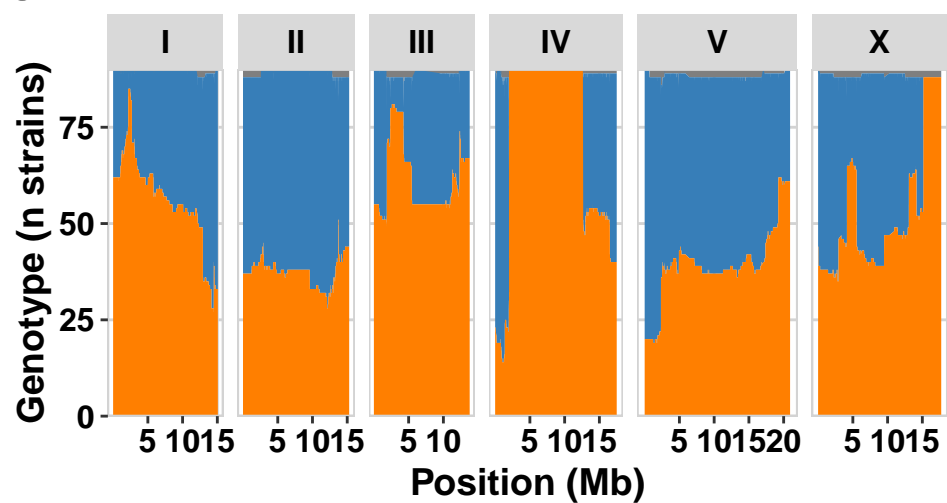

Supplement: Supplementary_figure1-Genetic_map_ddae148 [file supplementary_figure1-genetic_map_ddae148.pdf]

**A**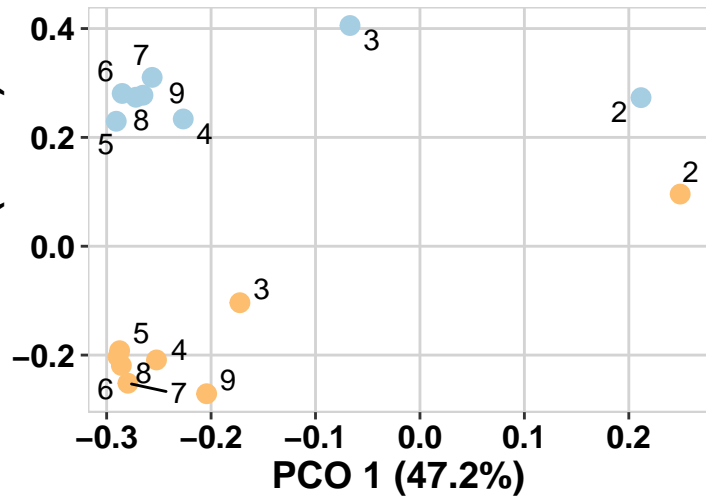**B**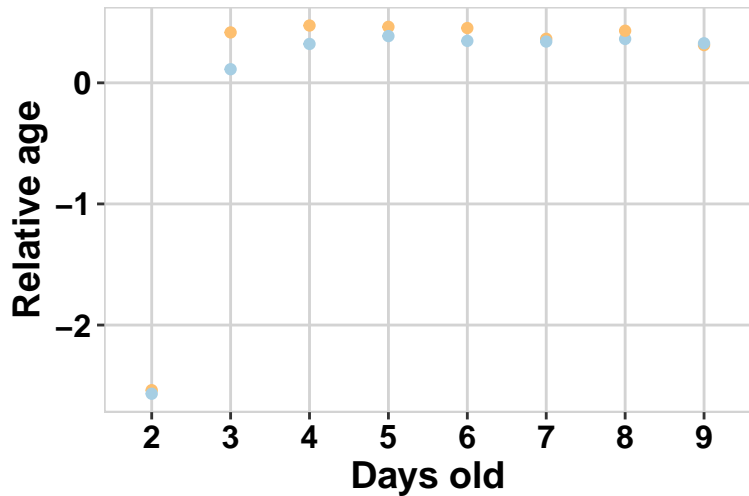

Supplement: Supplementary_figure2-transcriptomics_timeseries_ddae148 [file supplementary_figure2-transcriptomics_timeseries_ddae148.pdf]

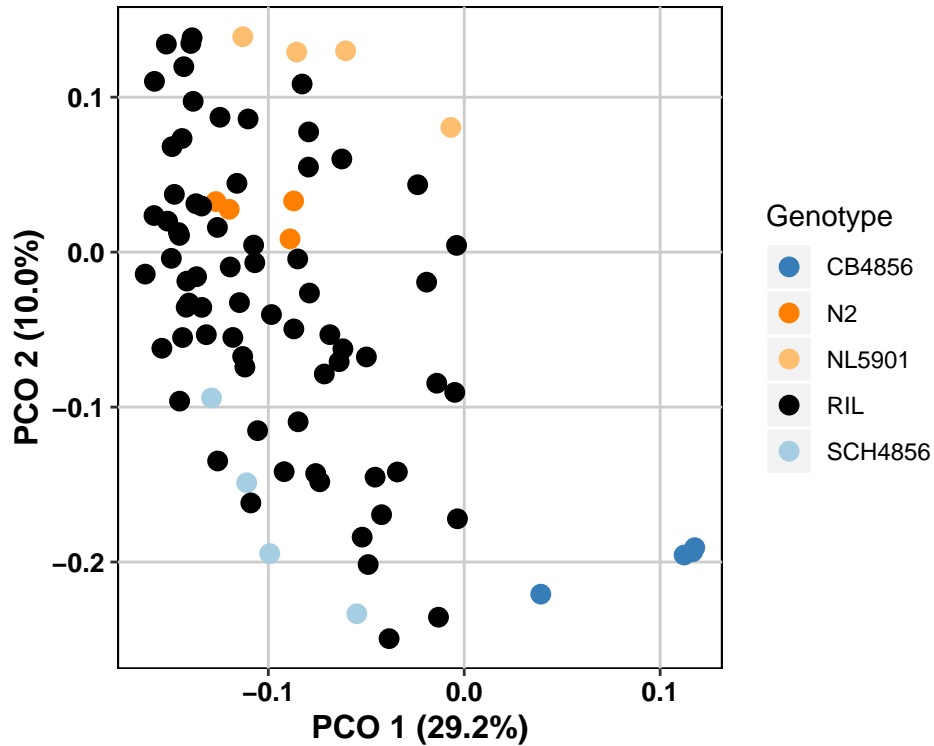

Supplement: Supplementary_figure3-PCA_RILs_ddae148 [file supplementary_figure3-pca_rils_ddae148.pdf]

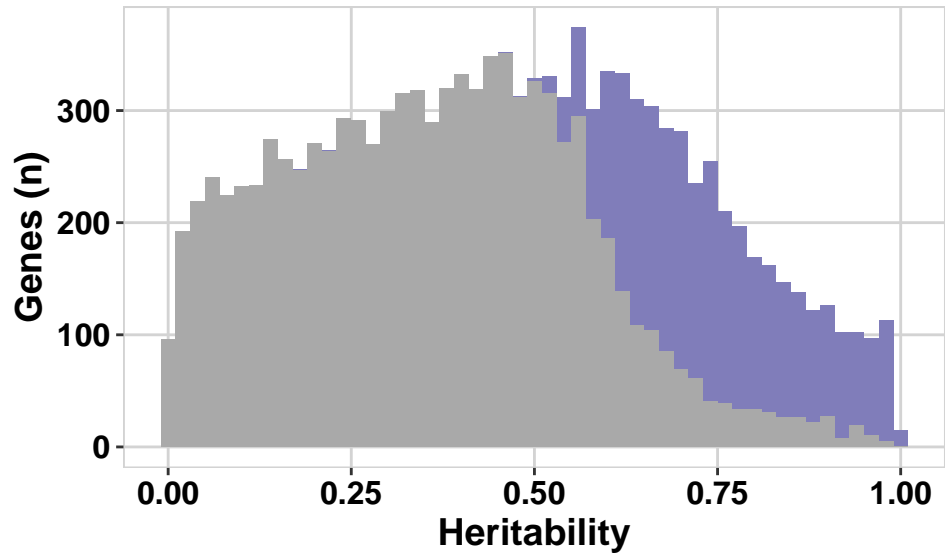

Supplement: Supplementary_figure4-Broad_sense_heritability_ddae148 [file supplementary_figure4-broad_sense_heritability_ddae148.pdf]

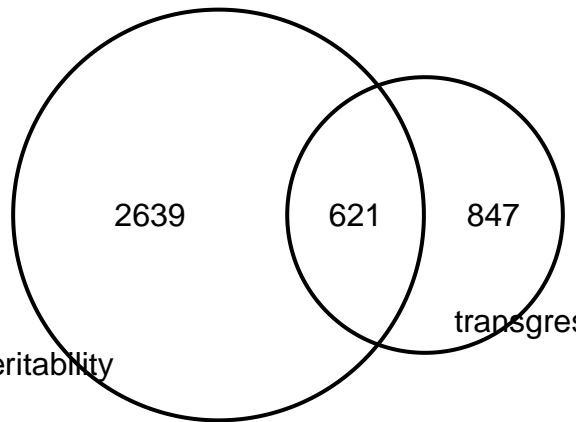

Supplement: Supplementary_figure5-Venn_ddae148 [file supplementary_figure5-venn_ddae148.pdf]

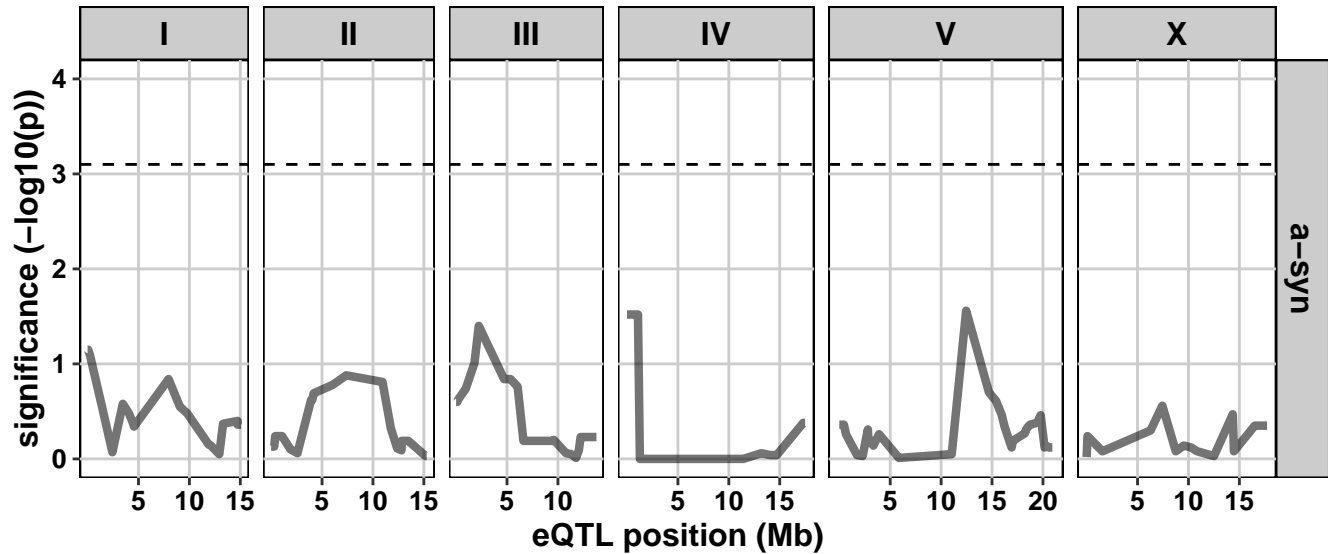

Supplement: Supplementary_figure6_qPCR_QTL_ddae148 [file supplementary_figure6_qpcr_qtl_ddae148.pdf]

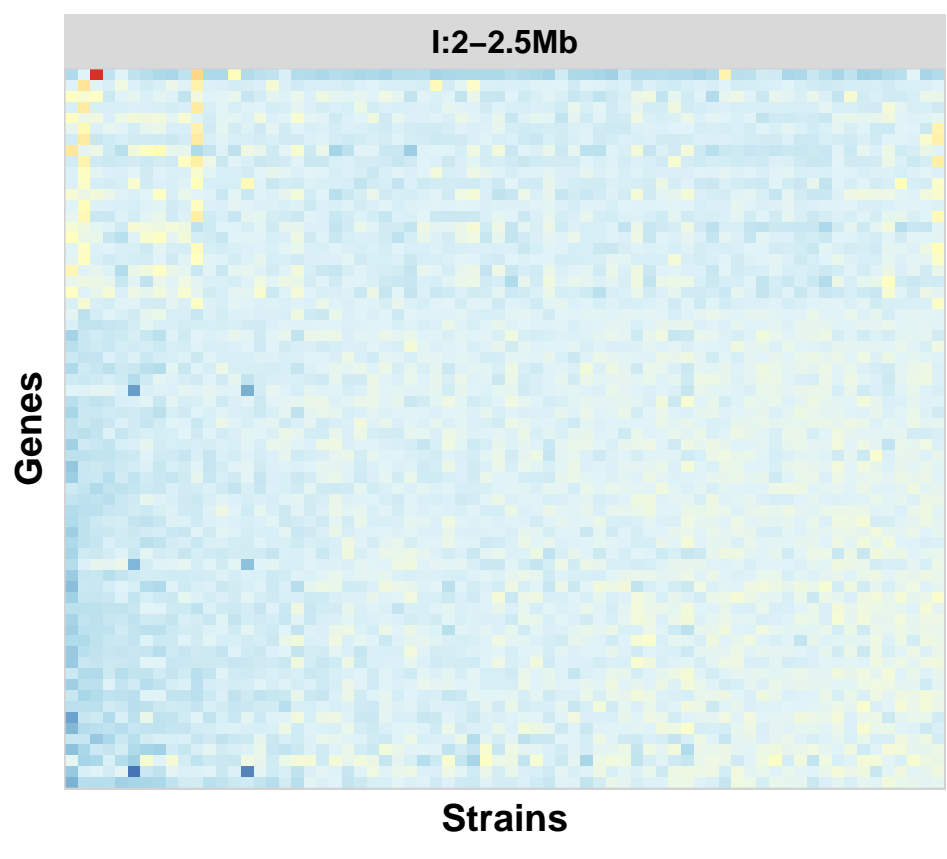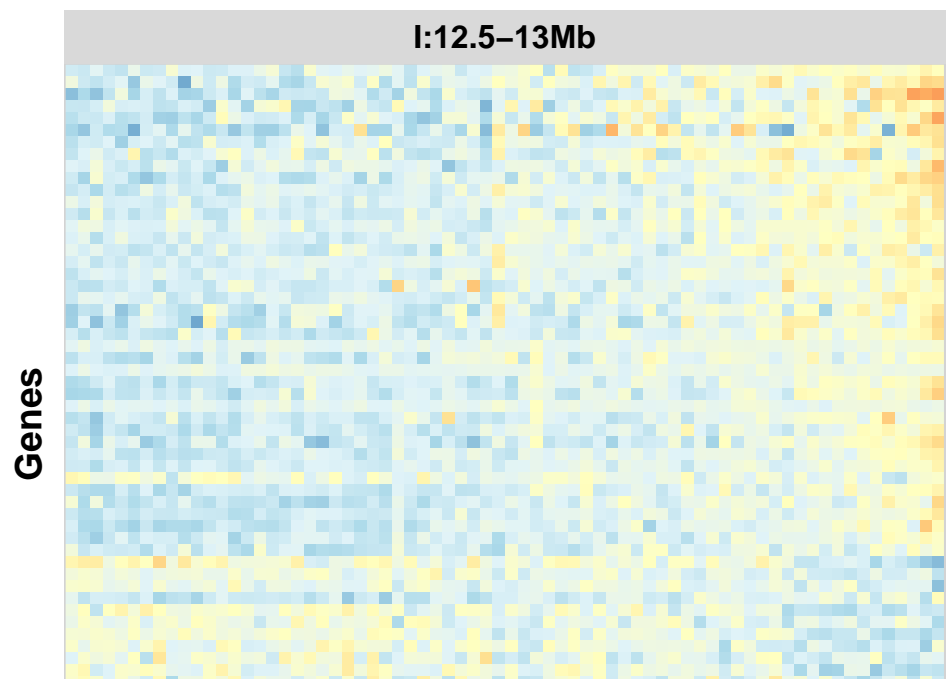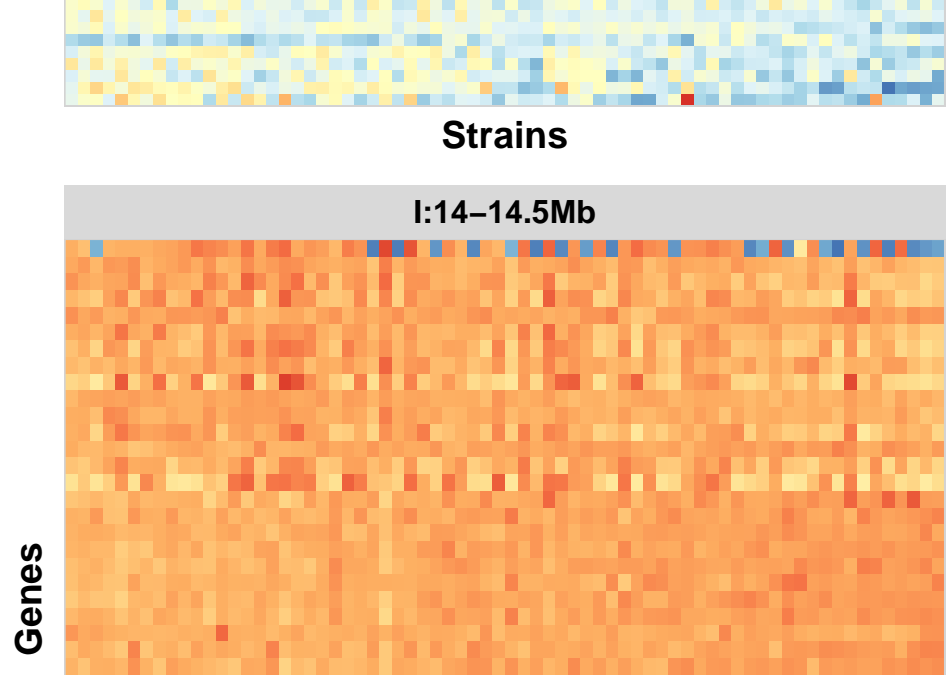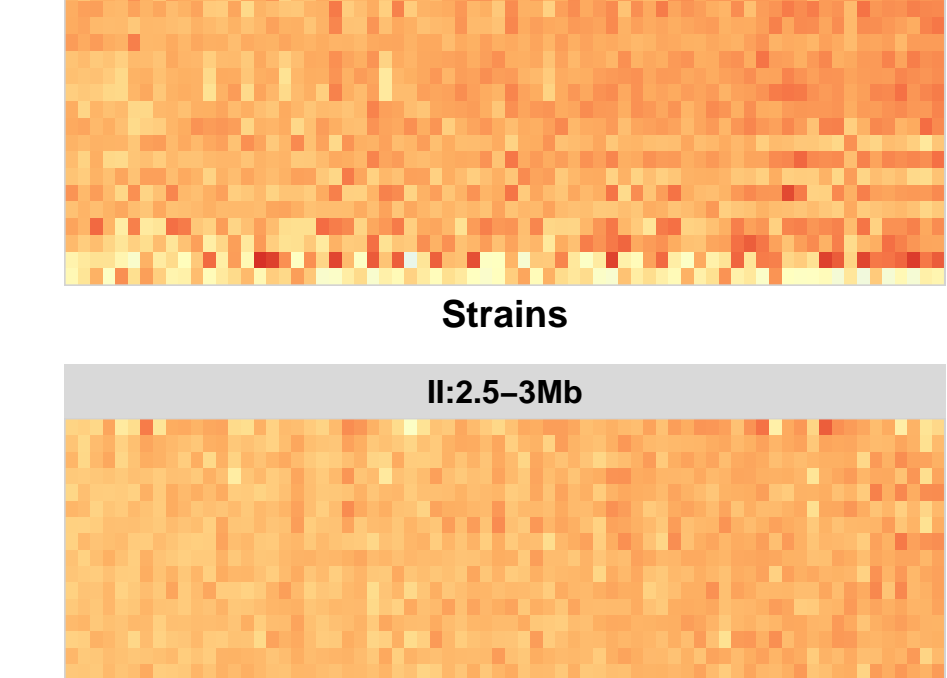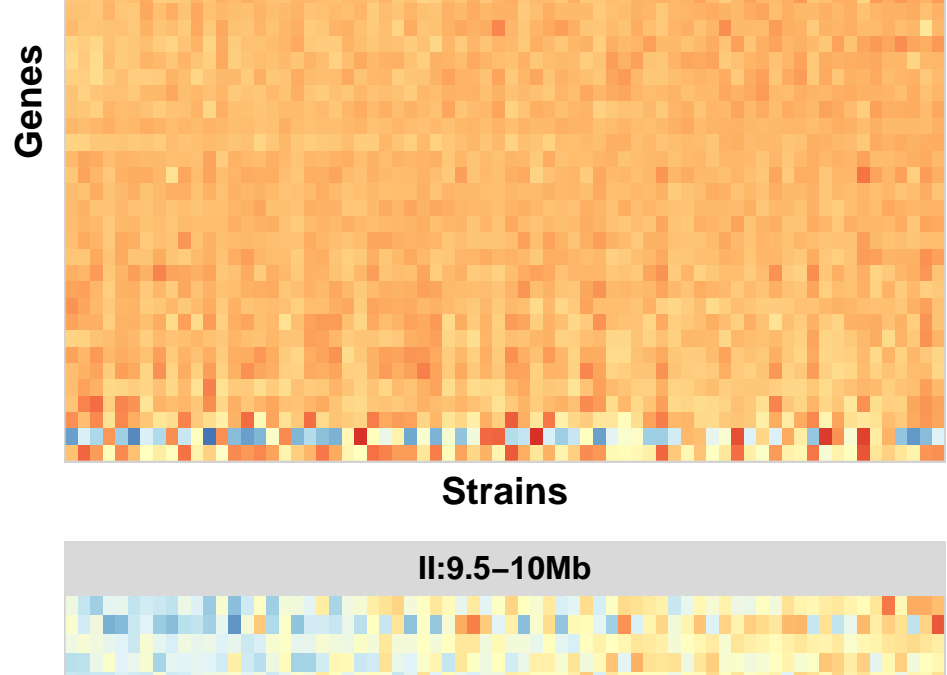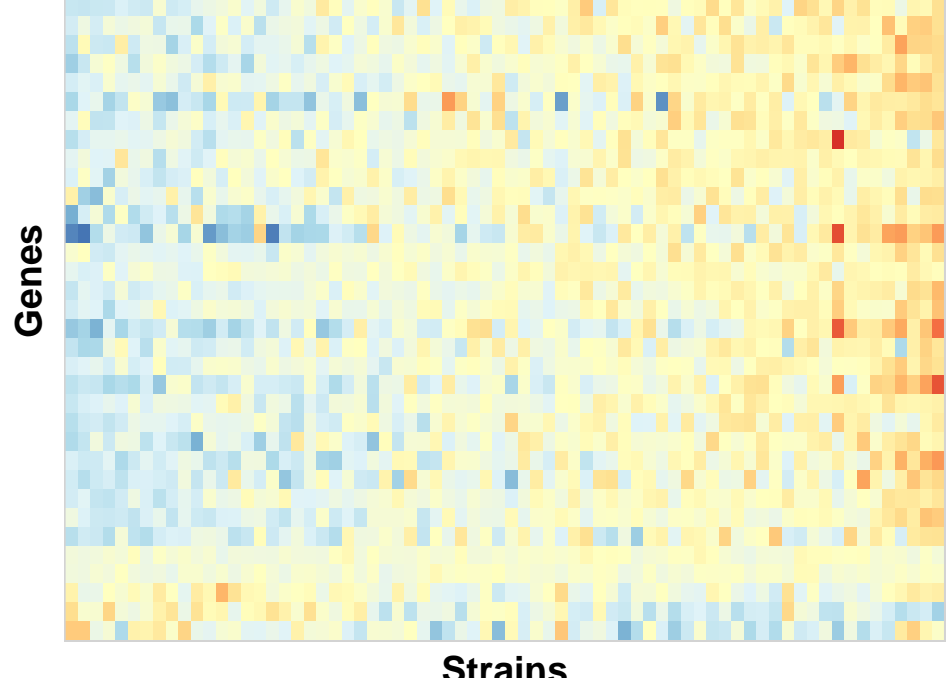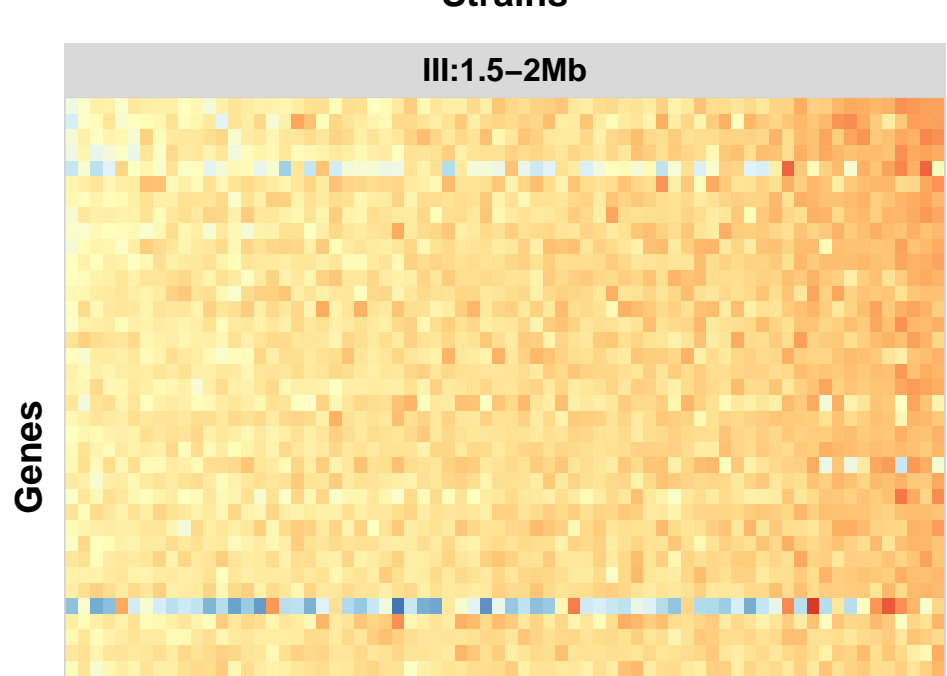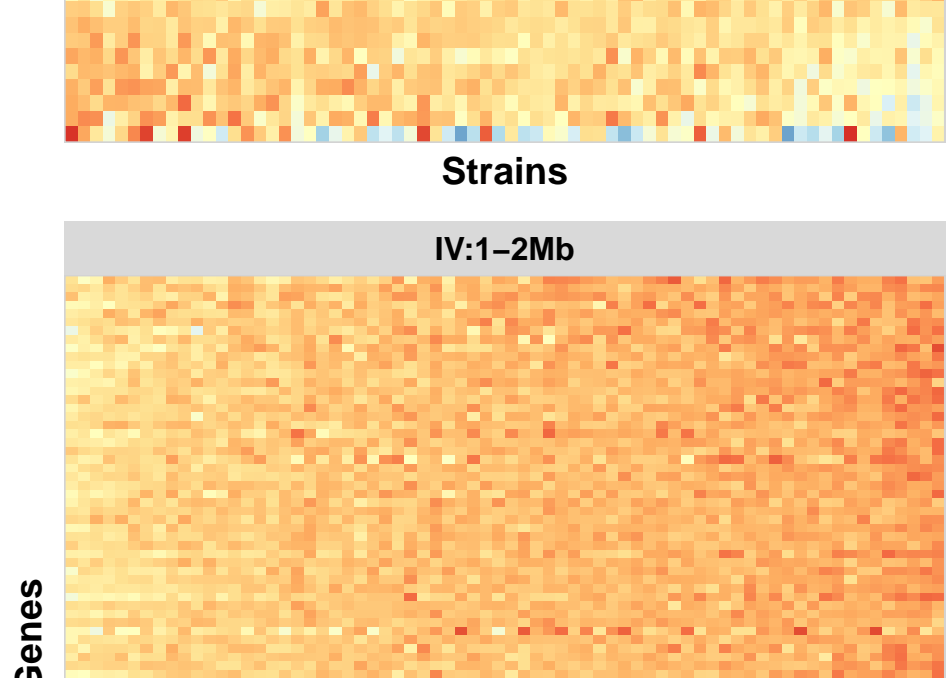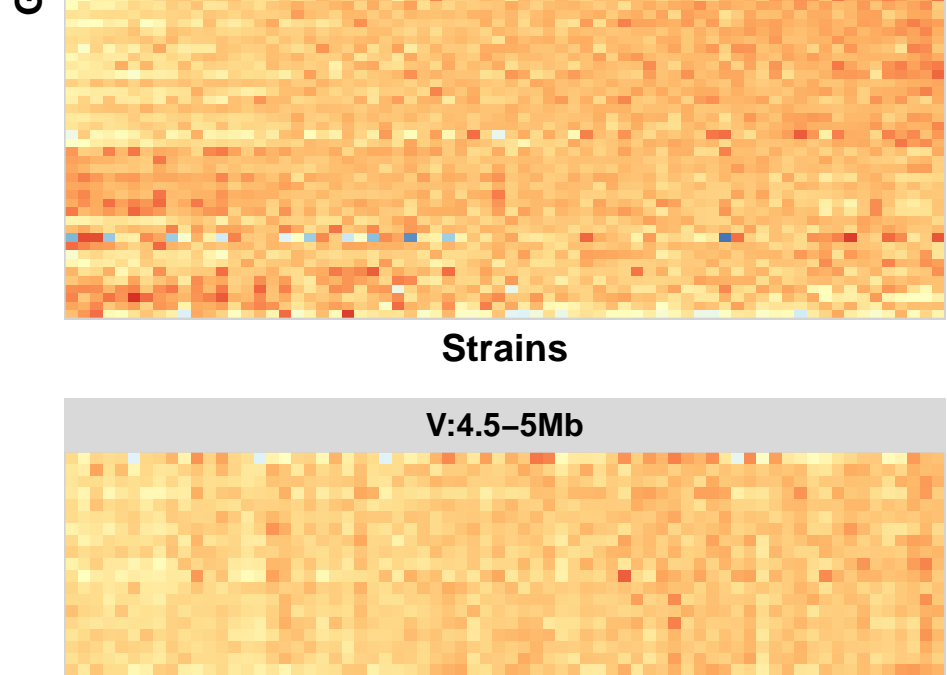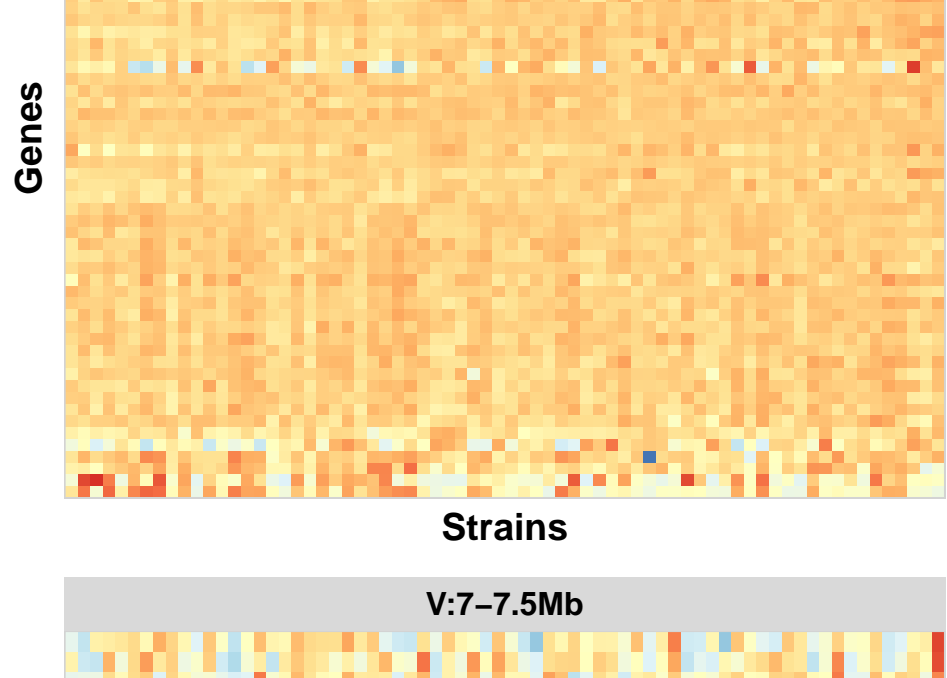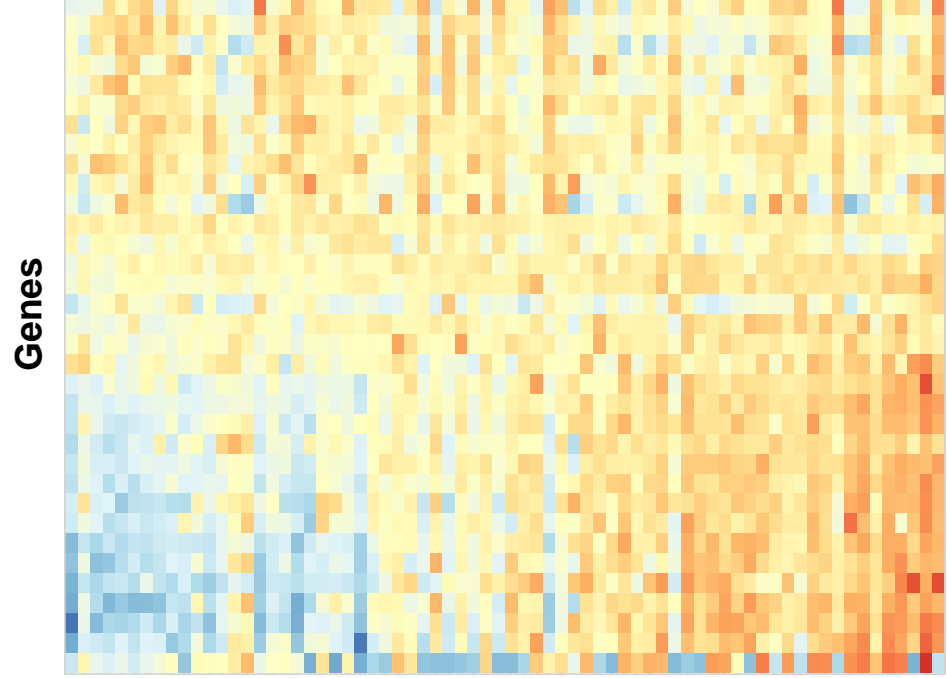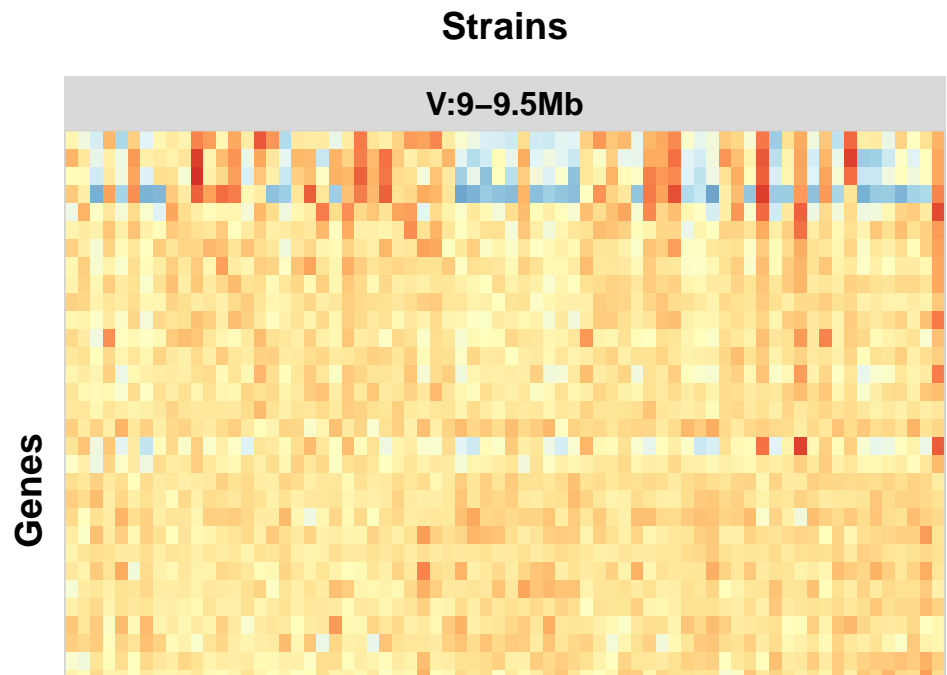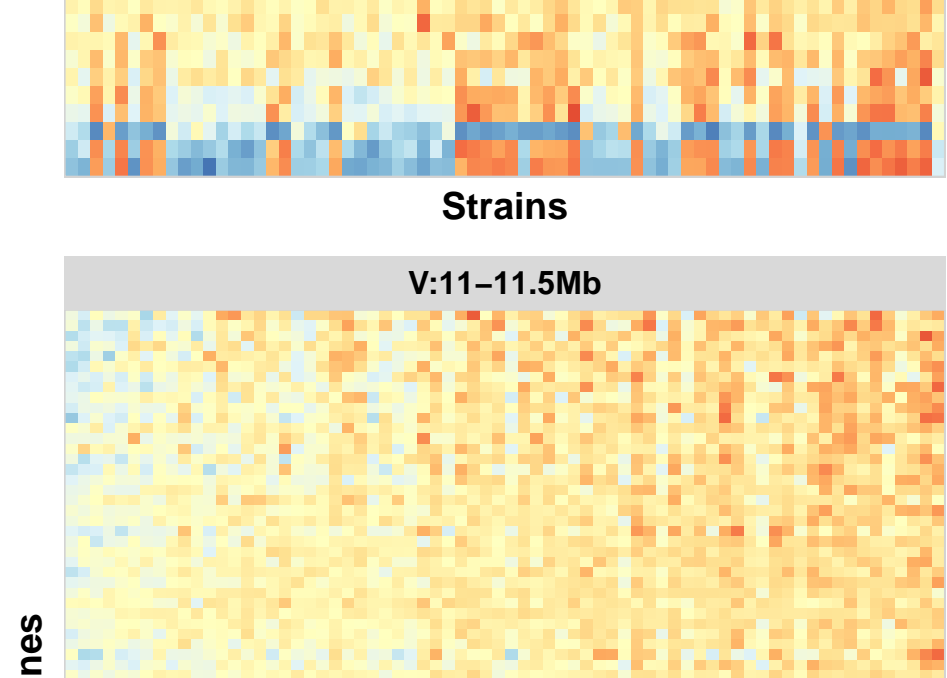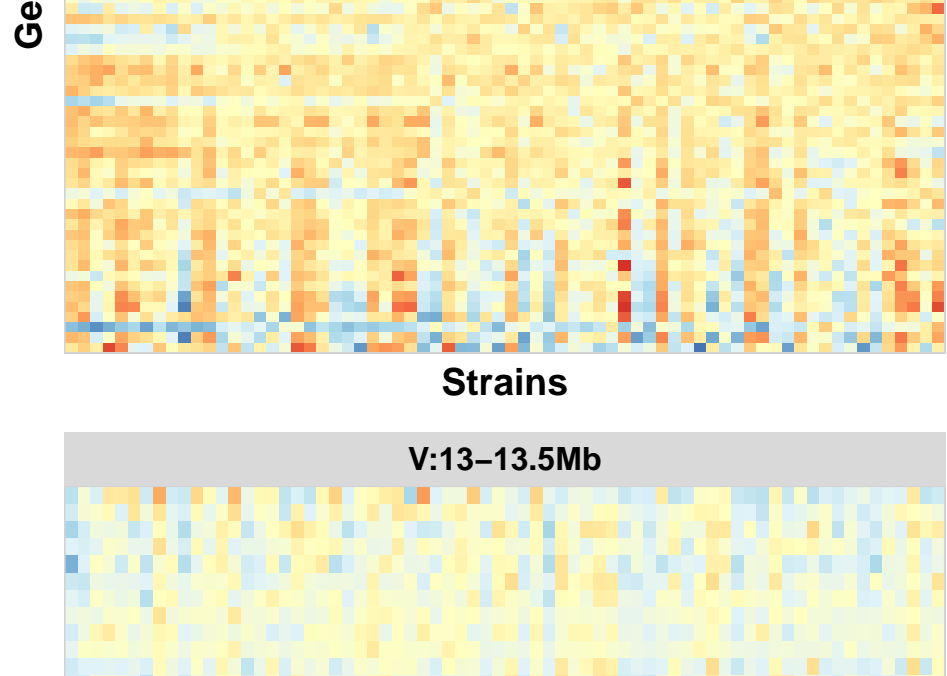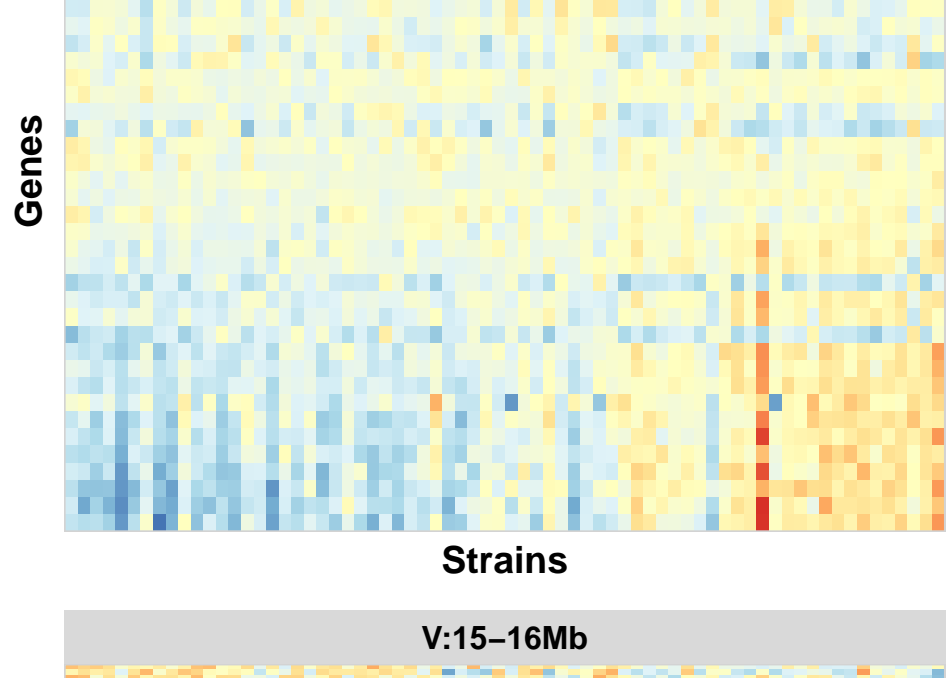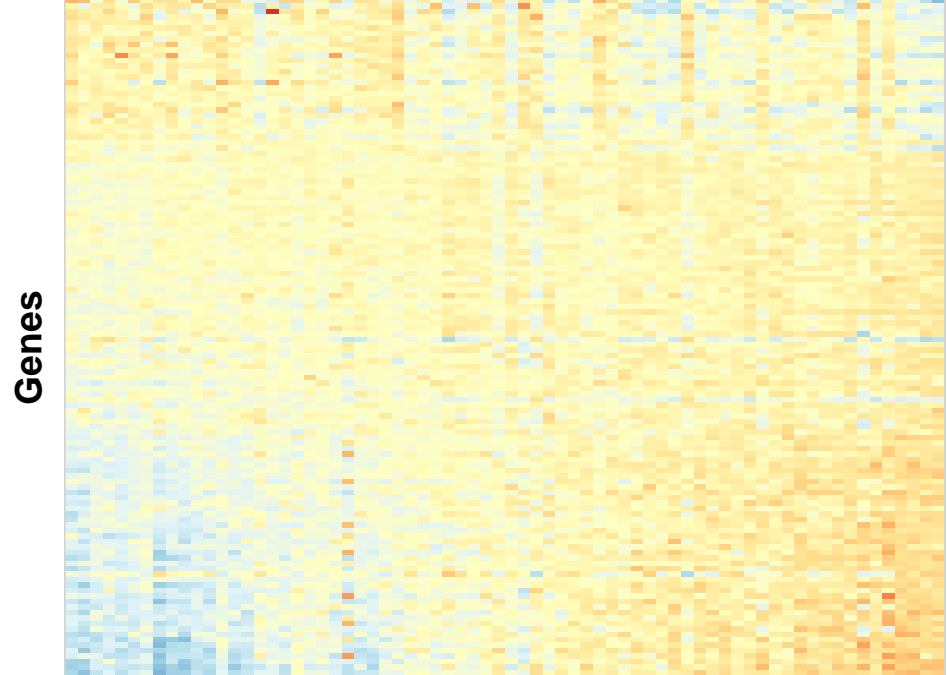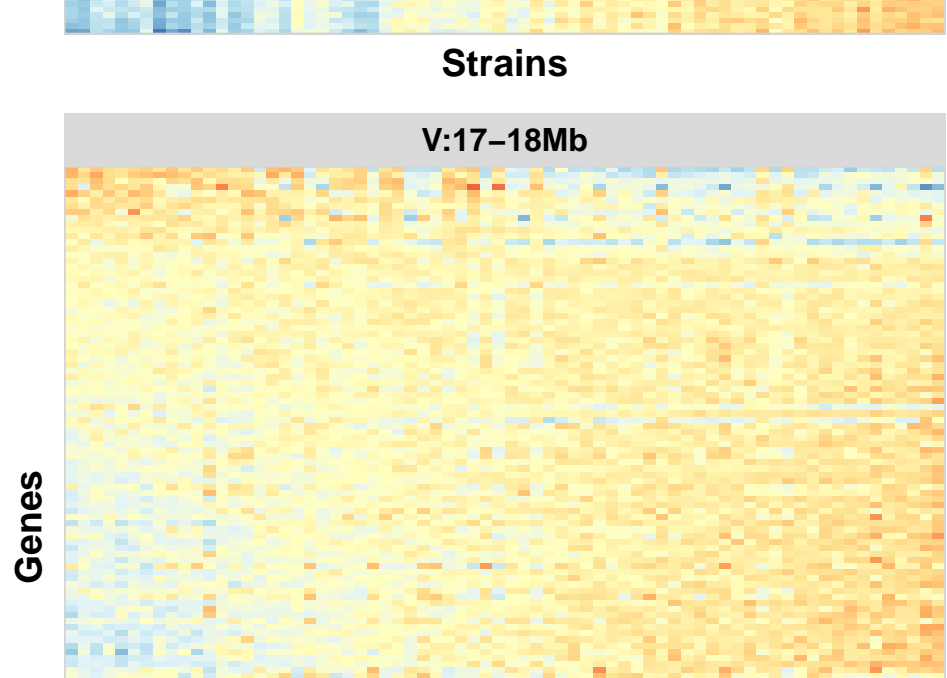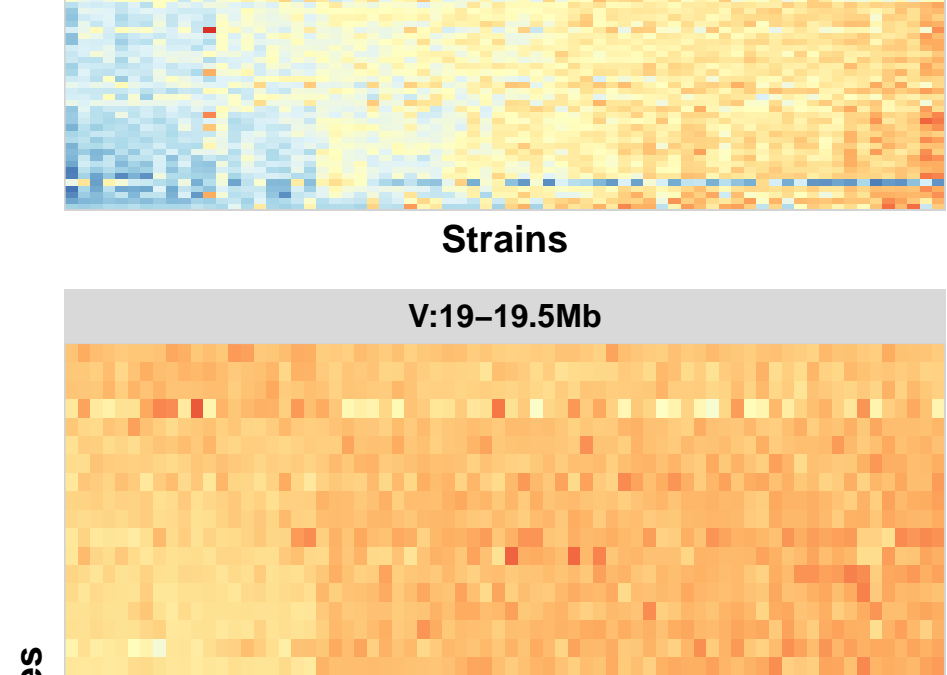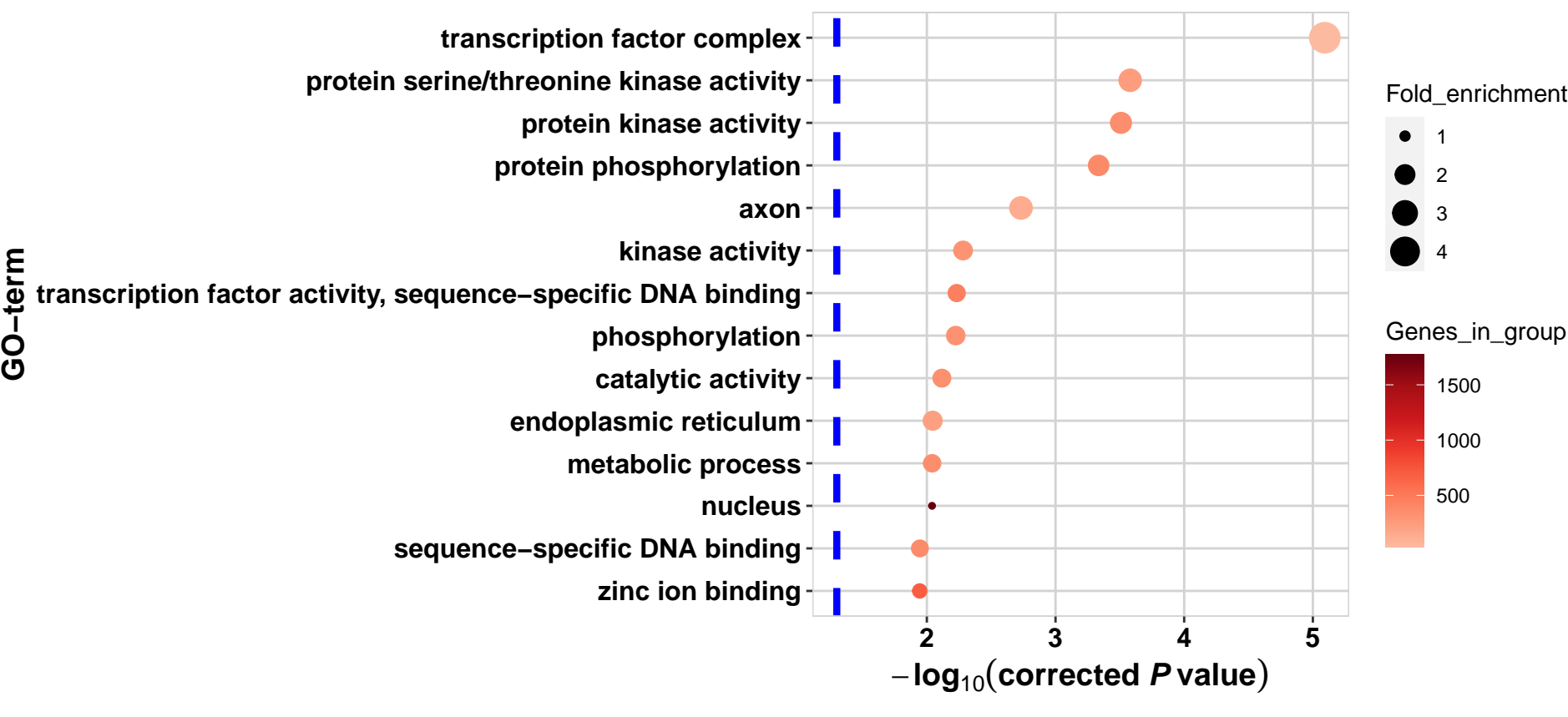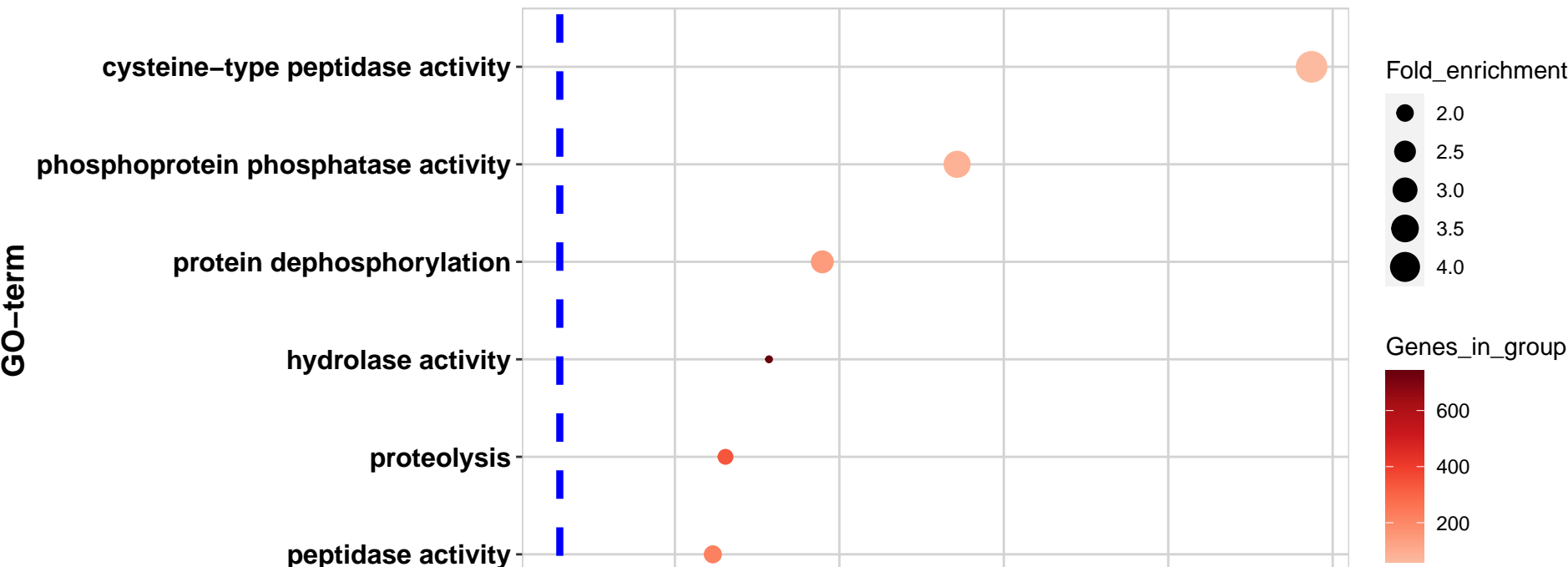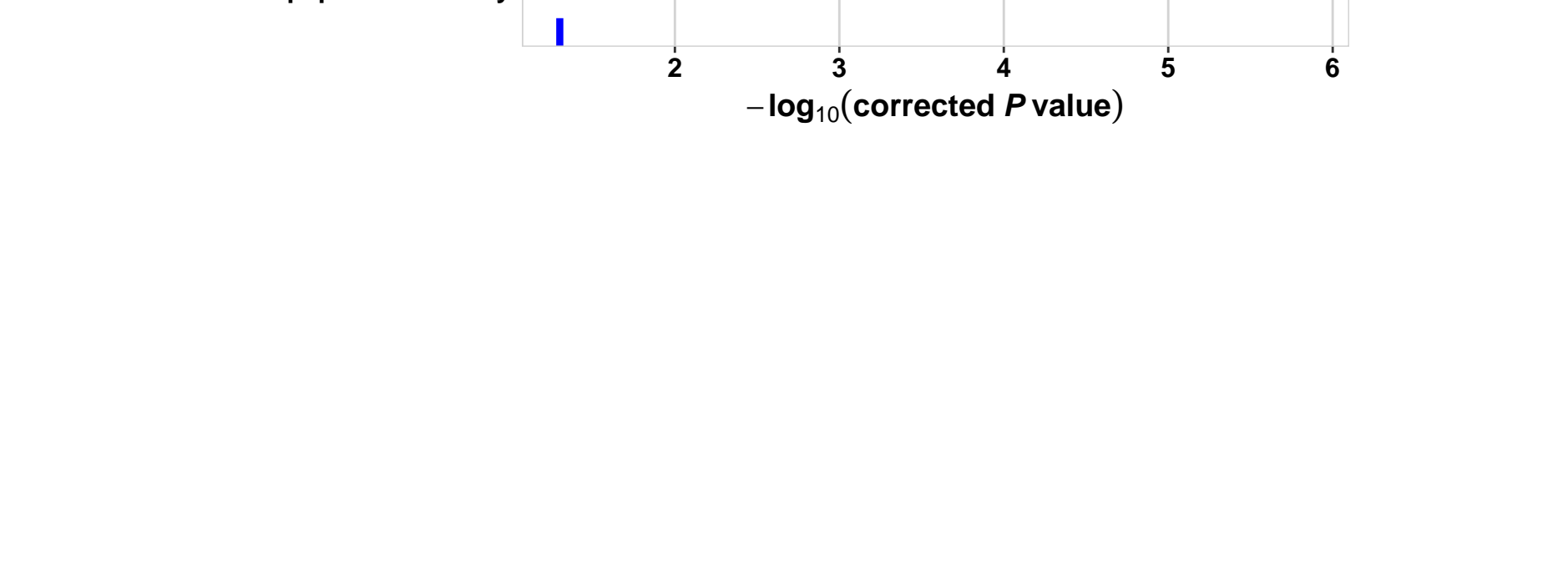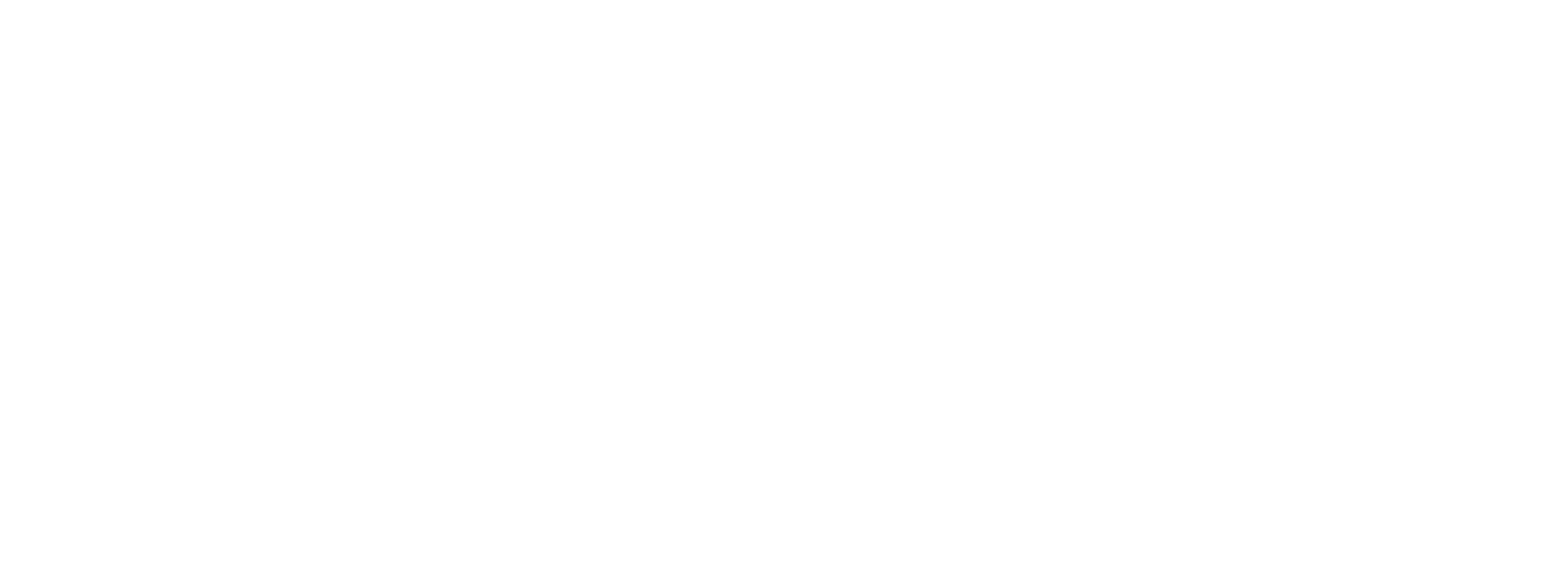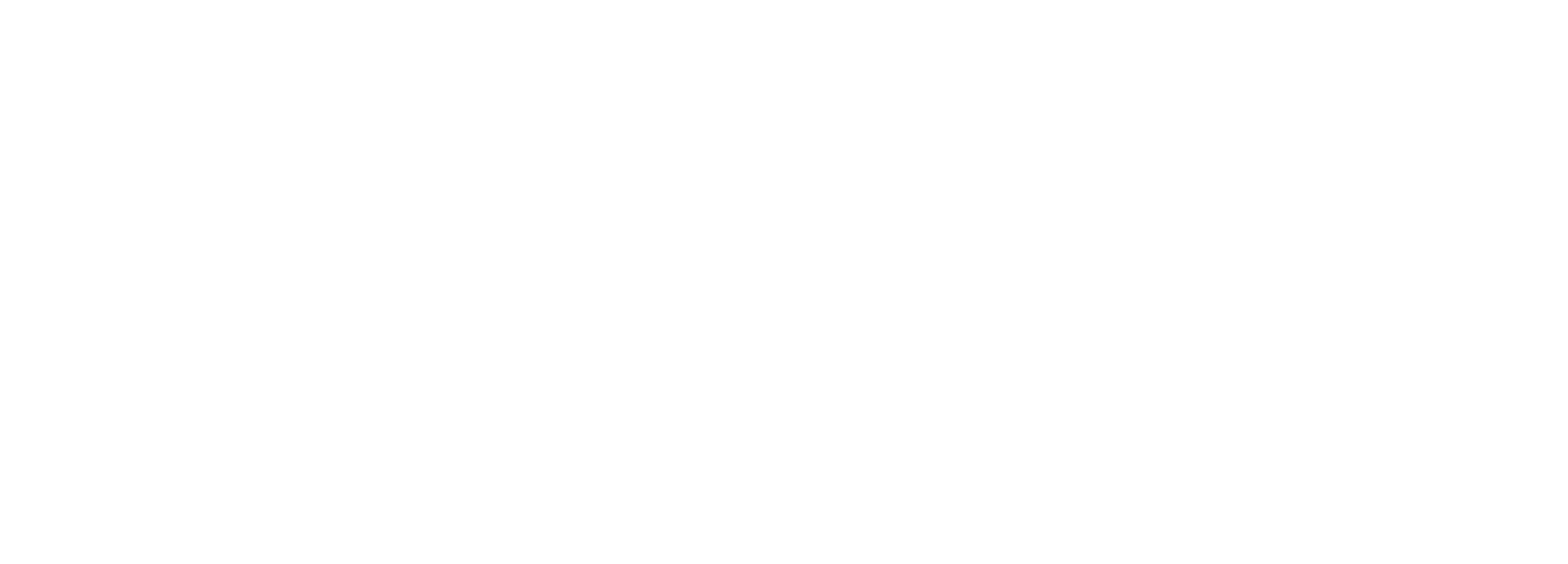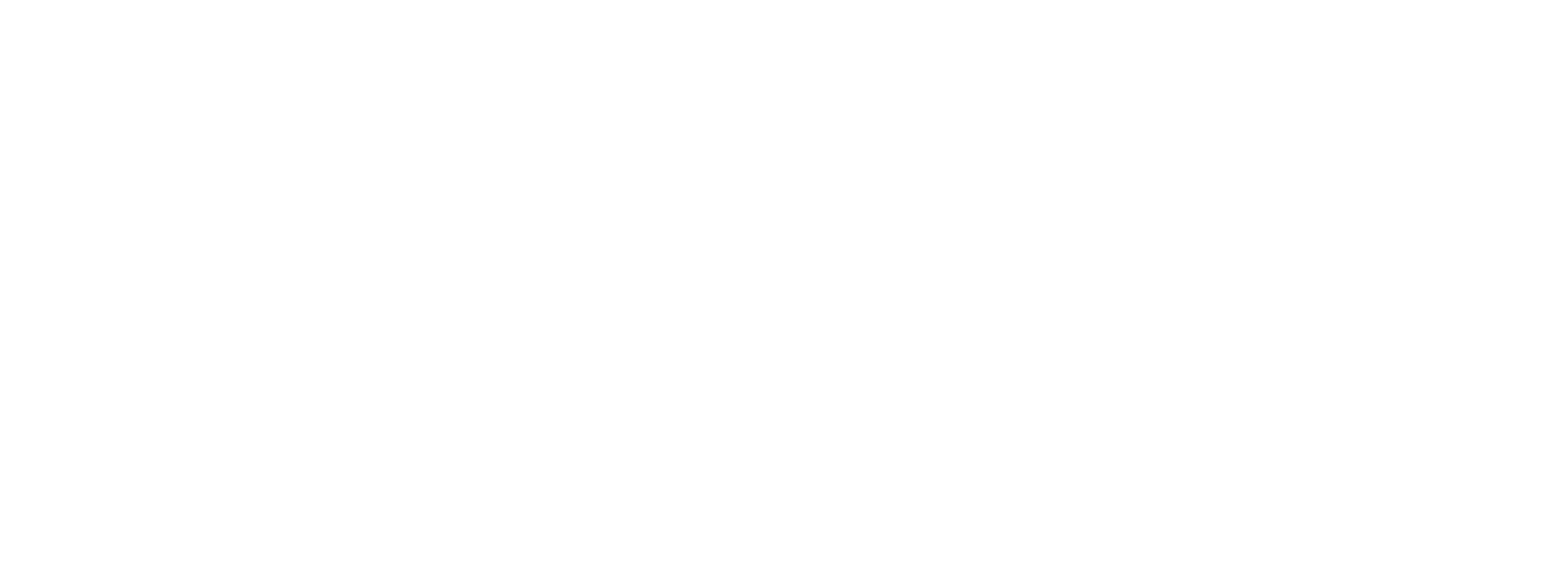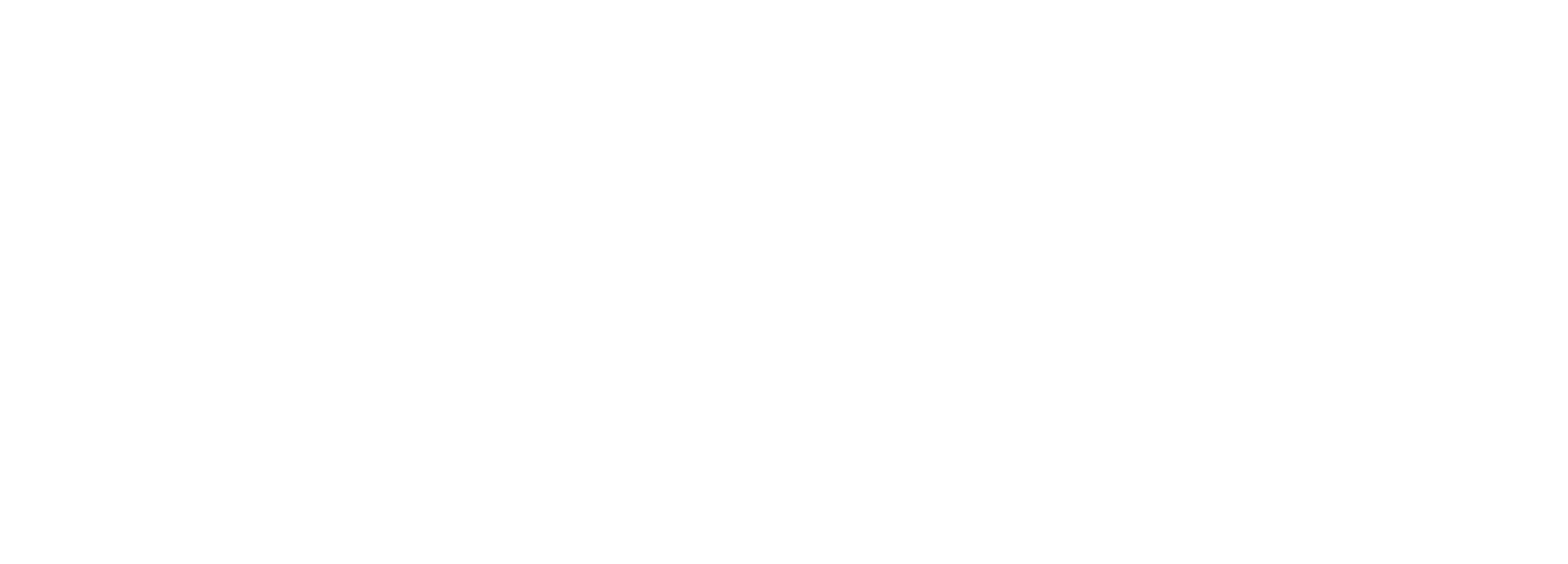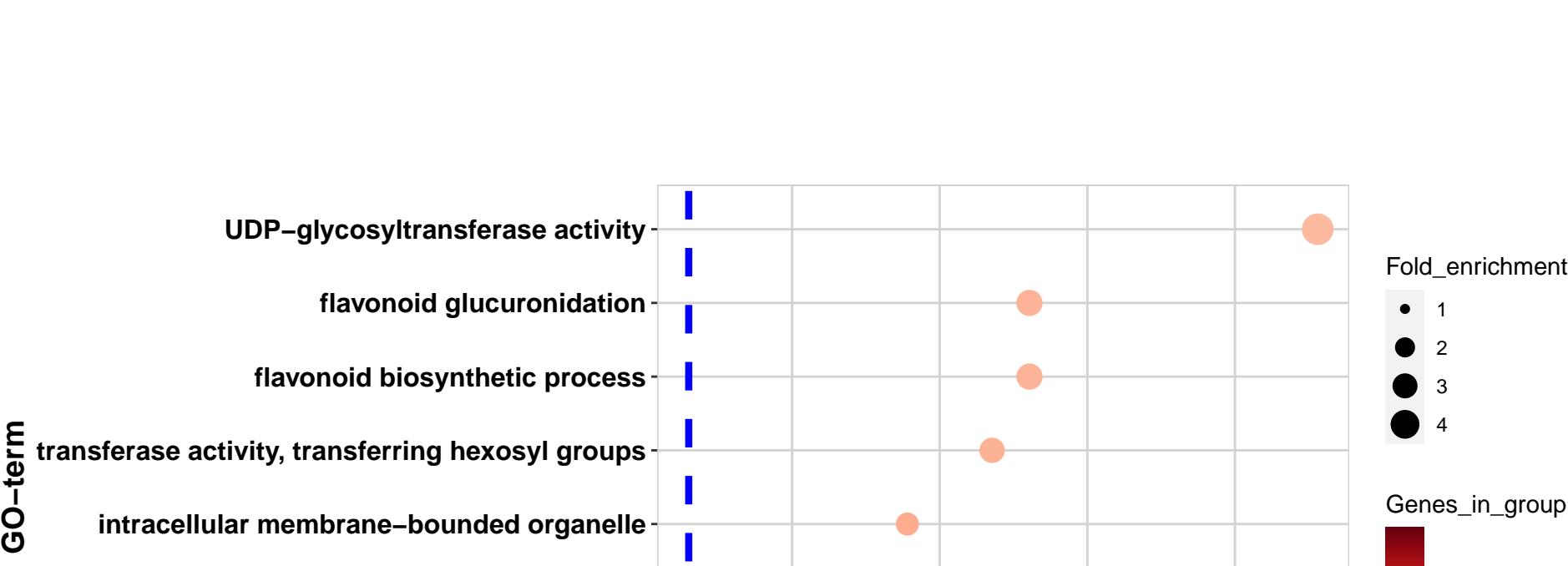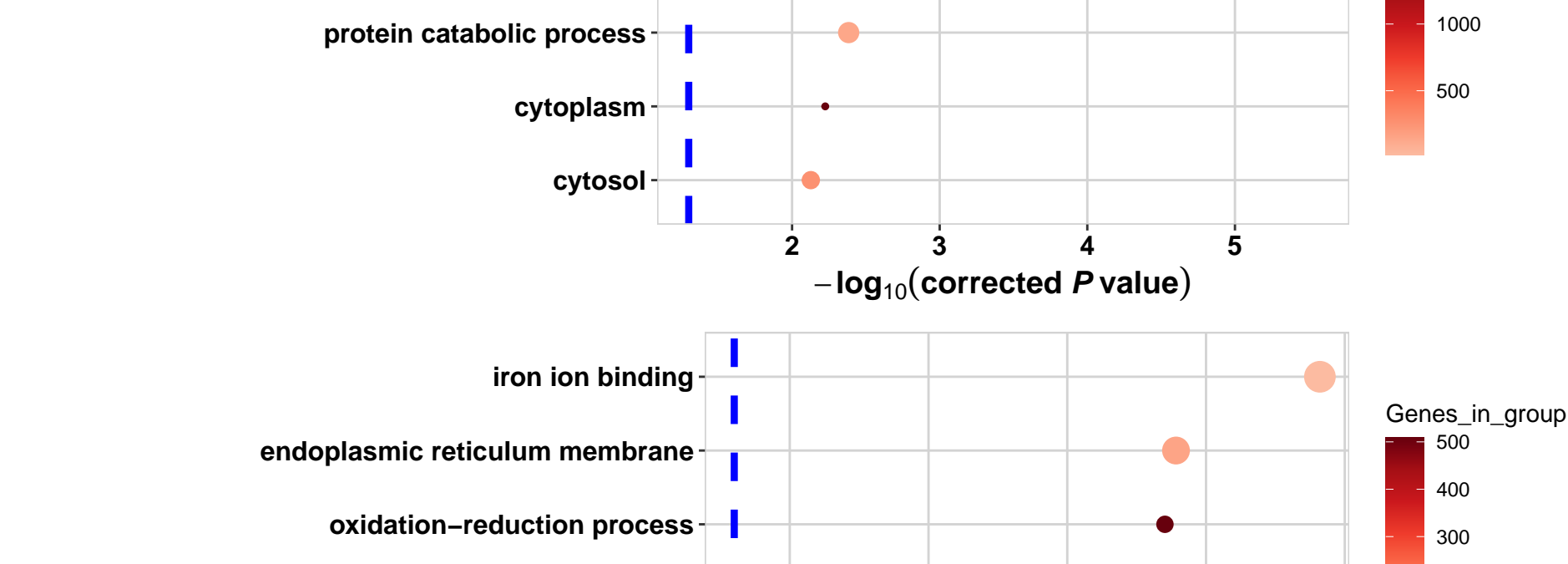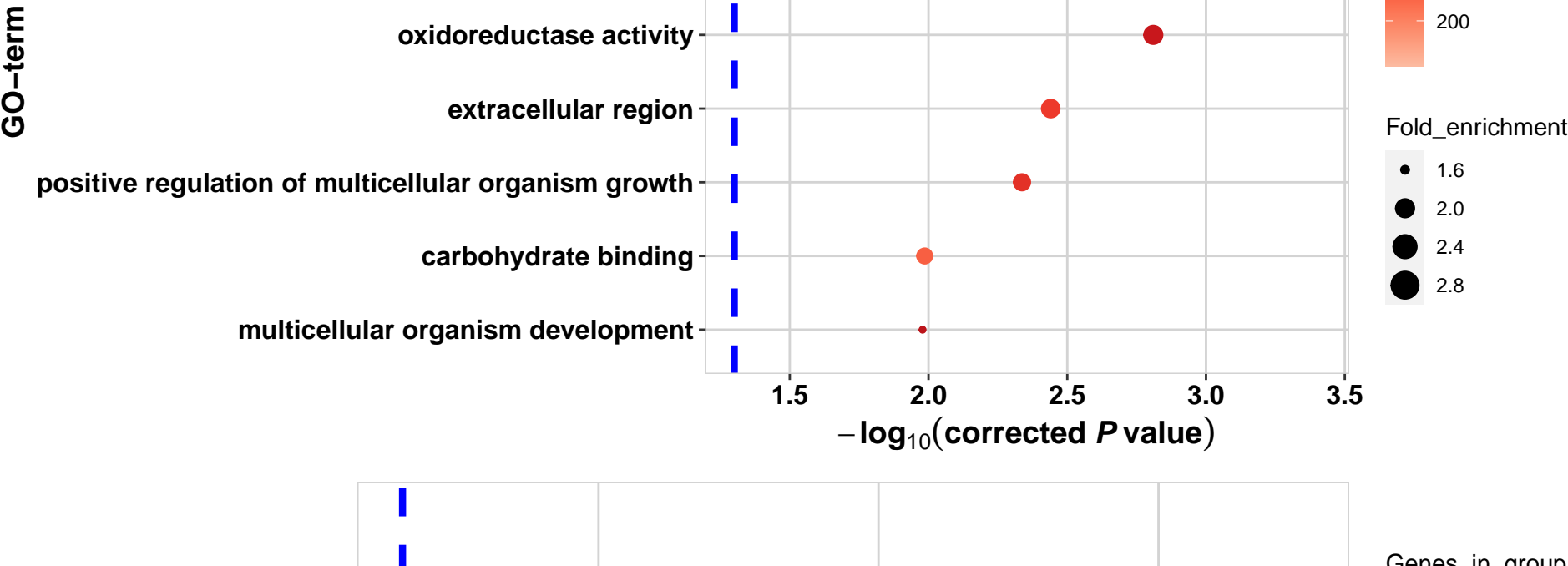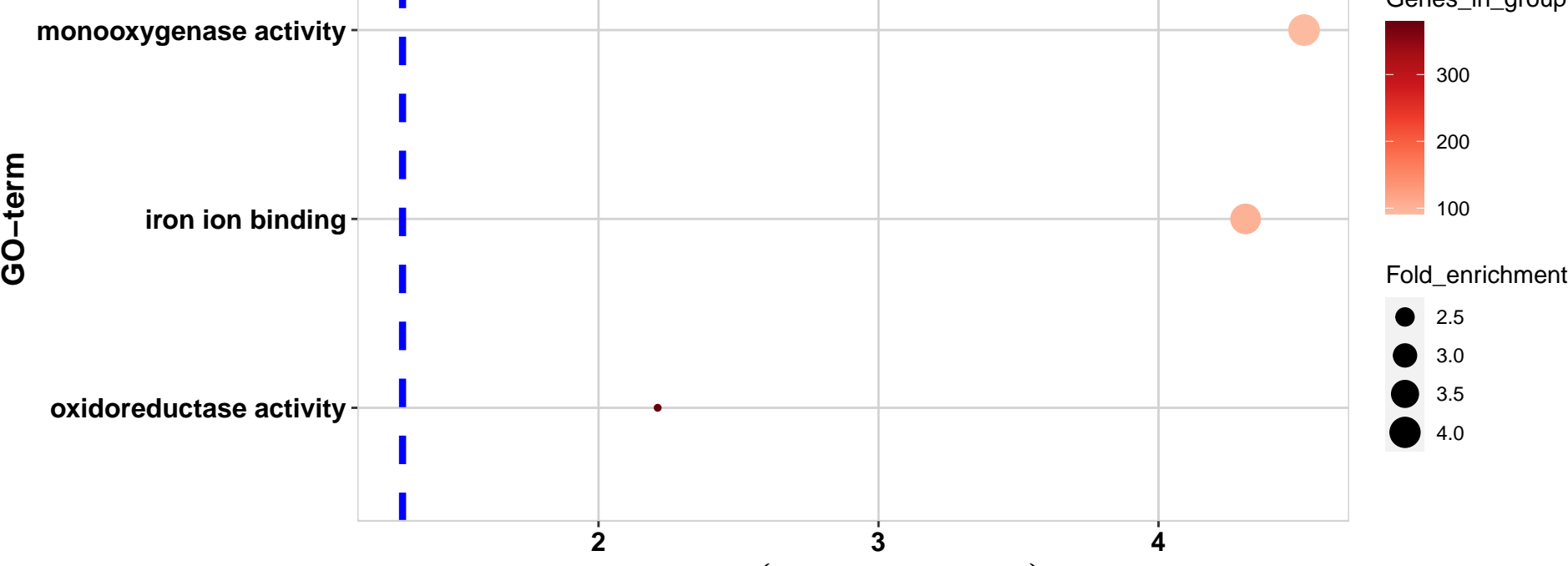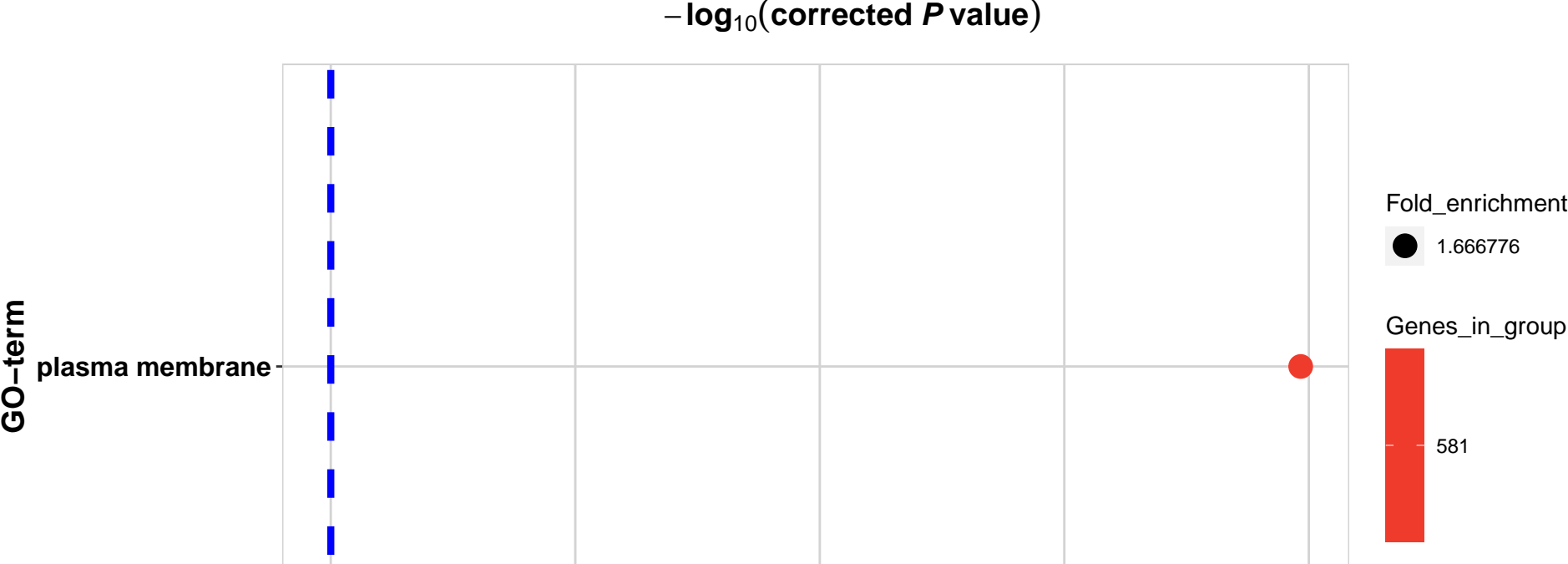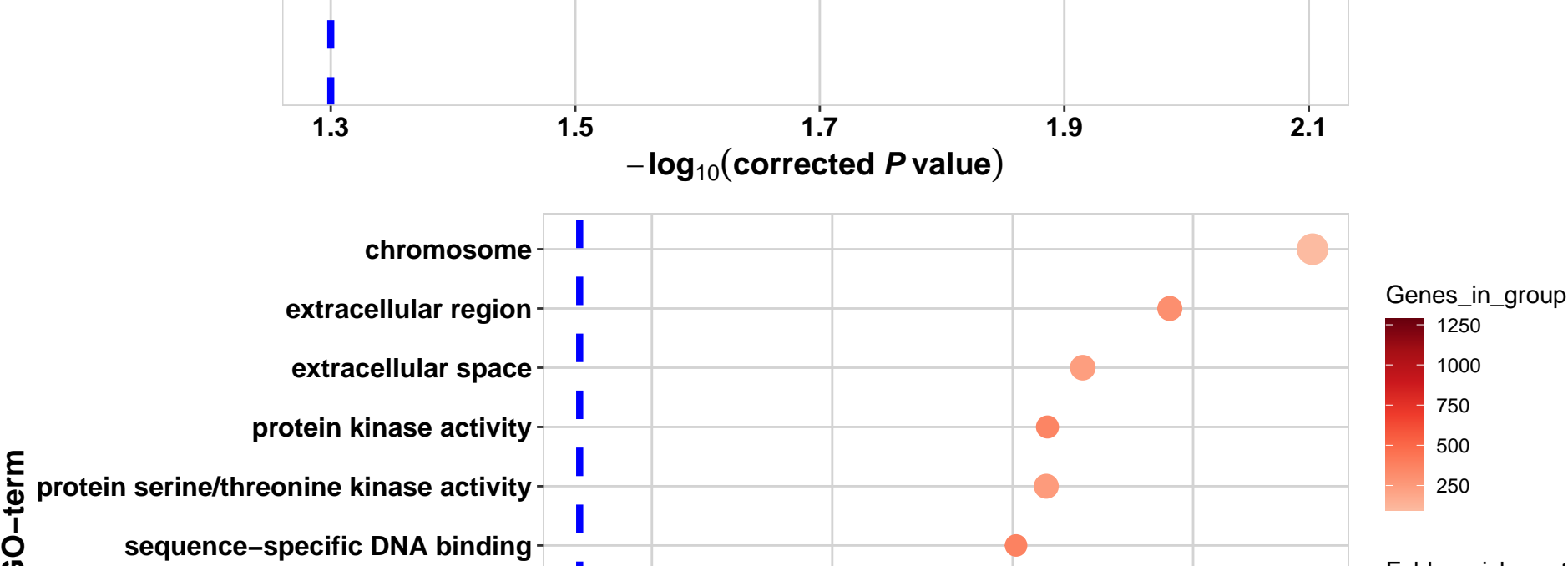

Supplement: Supplementary_figure7-Trans_band_enrichments_ddae148 [file supplementary_figure7-trans_band_enrichments_ddae148.pdf]

**A**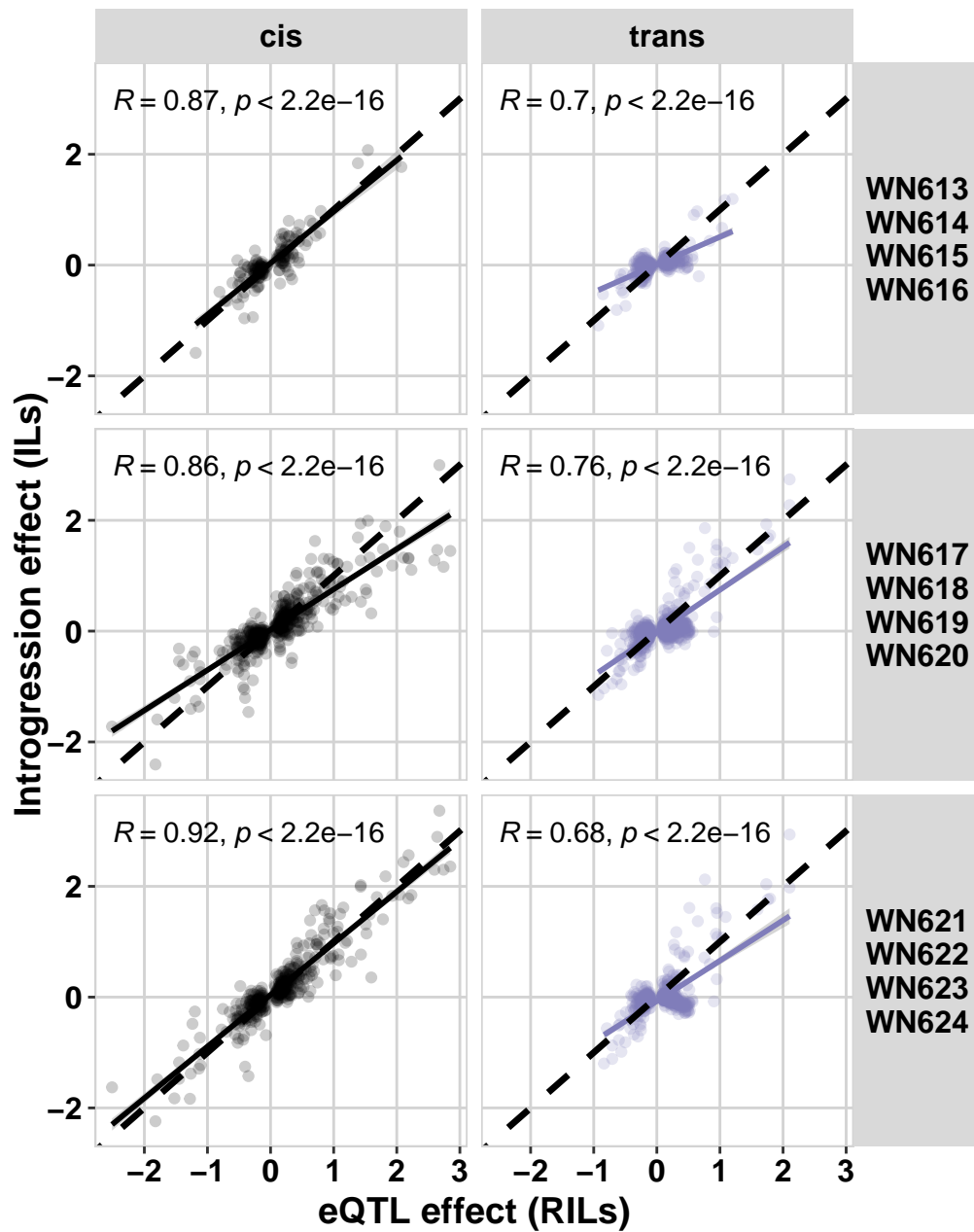**B**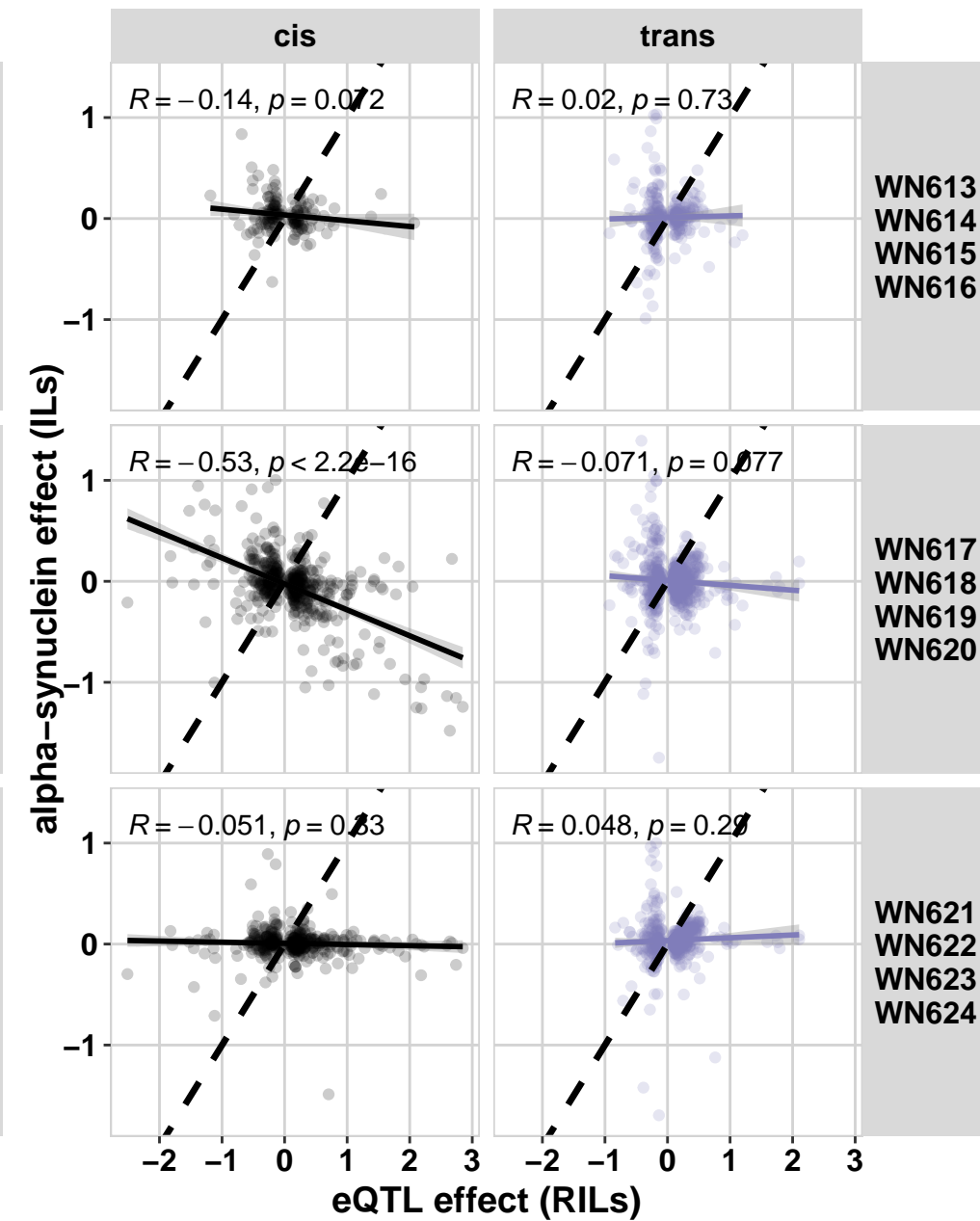**C**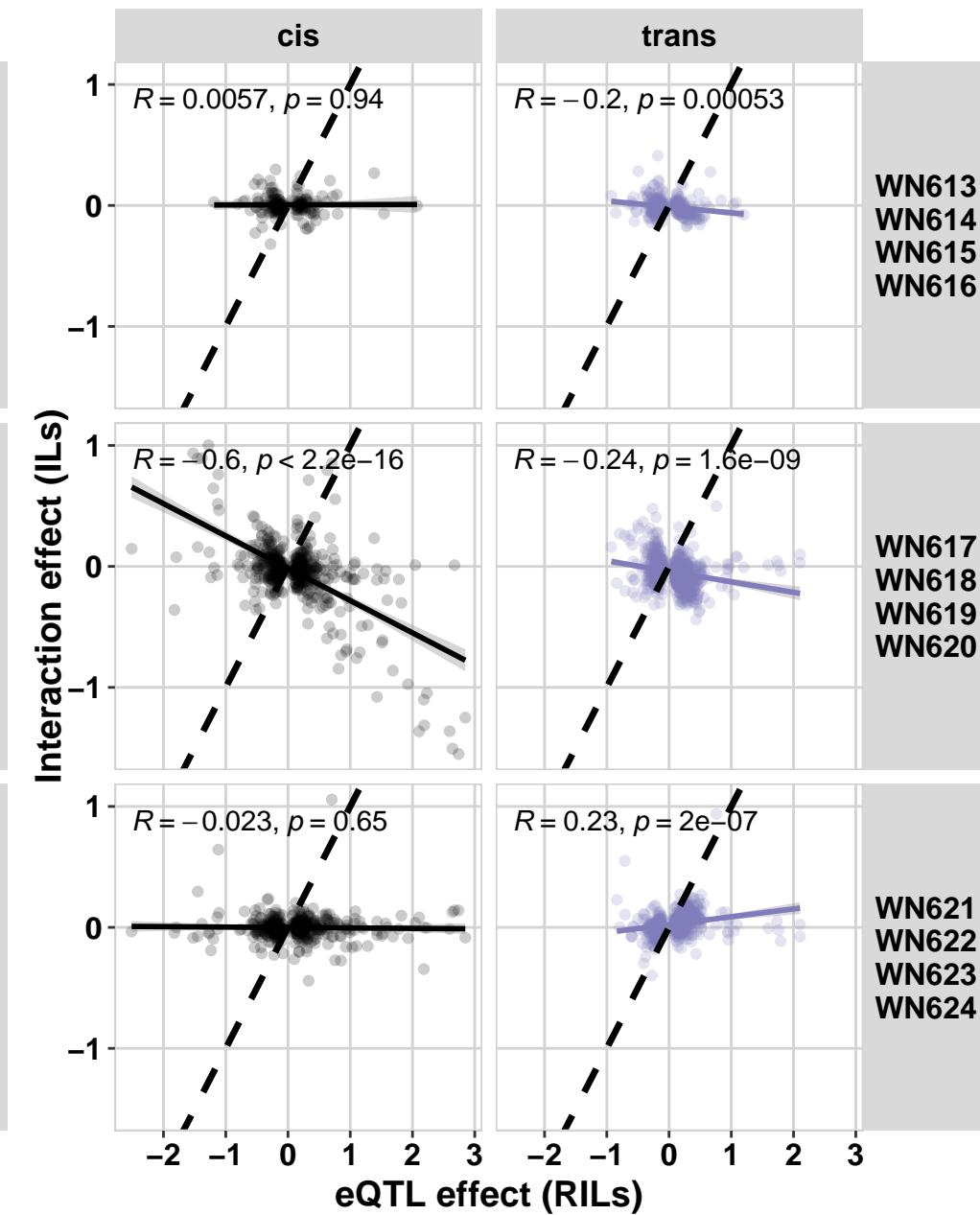

Supplement: Supplementary_figure8-IL_expression_eQTL_ddae148 [file supplementary_figure8-il_expression_eqtl_ddae148.pdf]
