# Supplementary material for: eQTL mapping in transgenic alpha-synuclein carrying Caenorhabditis elegans recombinant inbred lines
Source: Hum Mol Genet. 2024 Oct 23;33(24):2123–32. doi: 10.1093/hmg/ddae148 (PMC11630767; doi:10.1093/hmg/ddae148)
Supplement: Supplementary_Figure_legends_ddae148 [file supplementary_figure_legends_ddae148.docx]

**Supplementary Figure S1**: Genetic map and recombinant traits of αS RILs. (**A**) Complete genetic map of all 88 RILs created in this study. (**B**) Genetic and physical length of the markers at six chromosomes. (**C**) Genotype distribution of N2 and CB4856 genotype at six genotypes. Orange: N2, blue: CB4856.

**Supplementary Figure S2**: Time series experiment of NL5901 (orange) and SCH4856 (blue). (**A**) PCO analysis of NL5901 and SCH4856. The numbers indicate the age in days of the collected nematodes. (**B**) relative difference in age of NL5901 and SCH4856 based on gene expression of developmental genes.

**Supplementary Figure S3**: Genome-wide gene expression of the aS-RILs and their parental strains. A plot of a PCO analysis indicating variation in genome-wide gene expression. Orange: N2 and NL5901, blue: CB4856 and SCH4856, black: RILs.

**Supplementary Figure S4:** Heritability in the aS-RILs. A histogram of the estimated broad-sense heritability (H^2^) values for gene expression in the aS-RILs. The purple bars represent significantly heritable genes (permutation, FDR=0.05).

**Supplementary Figure S5:** Venn diagram of genes associated with heritability, transgression and eQTLs.

**Supplementary Figure S6:** α-synuclein mRNA expression phenotyping of αS RILs derived from NL5901×SCH4856. (**A**) α-synuclein mRNA expression in the RILs, Orange: NL5901, blue: SCH4856, grey: RILs. (**B**) eQTL mapping for α-synuclein mRNA expression, x-axis: marker position per chromosome (on top), y-axis: significance of association as -log_10_(p-value), dashed line: threshold, LOD>3.05.

**Supplementary Figure S7:** Visualization of the enrichment analysis of the identified *trans*-bands in the α-synuclein RIL panel. For each *trans­-*band the enrichment terms are shown including the number of genes, the fold enrichment and adjusted p-value for each specific enrichment.

**Supplementary Figure S8**: (**A**) Effects of CB4856 intogression lines correlated with the eQTL effects. (**B**) Effects correlated with the α-synuclein effect, and (**C**) correlated with the interaction effects between the introgression and the presence/absence of α-synuclein.

**Supplementary tables**: The legends for the supplementary tables can be found in the first tab of the .xlsx file.
